# Epigenetic Upregulation of MAGE-A Isoforms Promotes Breast Cancer Cell Aggressiveness

**DOI:** 10.3390/cancers13133176

**Published:** 2021-06-25

**Authors:** Chaeun Oh, Hwa-Ryeon Kim, Sumin Oh, Je Yeong Ko, Yesol Kim, Keunsoo Kang, Young Yang, Jongmin Kim, Jong Hoon Park, Jae-Seok Roe, Kyung Hyun Yoo

**Affiliations:** 1 Laboratory of Biomedical Genomics, Department of Biological Sciences, Sookmyung Women’s University, Seoul 04310, Korea; codns2751@sookmyung.ac.kr (C.O.); soh@sookmyung.ac.kr (S.O.); 2 Department of Biochemistry, College of Life Science and Biotechnology, Yonsei University, Seoul 03722, Korea; kim.hy@yonsei.ac.kr; 3 Research Institute of Women’s Health, Sookmyung Women’s University, Seoul 04310, Korea; 4 Department of Biological Sciences, Sookmyung Women’s University, Seoul 04310, Korea; jeyeong@sookmyung.ac.kr (J.Y.K.); yesol.kim89@sookmyung.ac.kr (Y.K.); yyang@sookmyung.ac.kr (Y.Y.); jkim@sookmyung.ac.kr (J.K.); parkjh@sookmyung.ac.kr (J.H.P.); 5 Department of Microbiology, College of Science & Technology, Dankook University, Cheonan 31116, Korea; kangk1204@gmail.com

**Keywords:** molecular subtype, breast cancer, MAGEA12, chromatin modification

## Abstract

**Simple Summary:**

Breast cancer is a heterogeneous disease that has complex causes and mechanisms of development. Currently, patient treatment options depend on the breast cancer molecular subtype, which is classified based on the presence or absence of hormone receptors and HER2. However, this classification system has limitations in terms of predicting responsiveness to anticancer drugs and patient outcomes. In this study, we present a new approach to classifying molecular breast cancer subtypes: it is based on changes in histone modifications in the promoter region of the MAGEA12 locus, which we found related closely to MAGEA12 expression and MAGEA12-associated malignancy of breast cancer cells.

**Abstract:**

After decades-long efforts to diagnose and treat breast cancer, the management strategy that has proved most successful to date is molecular-subtype-specific inhibition of the hormone receptors and HER2 that are expressed by individual cancers. Melanoma-associated antigen (MAGE) proteins comprise >40 highly conserved members that contain the MAGE homology domain. They are often overexpressed in multiple cancers and contribute to cancer progression and metastasis. However, it remains unclear whether the biological activity arising from MAGE gene expression is associated with breast cancer subtypes. In this study, we analyzed the RNA-sequencing (RNA-seq) data of 70 breast cancer cell lines and found that MAGEA12 and MAGEA3 were highly expressed in a subset of these lines. Significantly, MAGEA12 and MAGEA3 expression levels were independent of hormone receptor expression levels but were closely associated with markers of active histone modifications. This indicates that overexpression of these genes is attributable to epigenetic deregulation. RNA-seq of MAGEA12-depleted cells was then used to identify 382 candidate targets of MAGEA12 that were downregulated by MAGEA12 depletion. Furthermore, our gain-of-function experiments showed that MAGEA12 overexpression promoted aggressive behaviors of malignant breast cancer cells, including enhancing their cell migration and invasion. These changes were associated with increased epigenetic deregulation of the MAGEA12 signature genes. Thus, MAGEA12 may play an important role in breast cancer malignancy. Taken together, our findings suggest that MAGEA12 could be a promising therapeutic target in breast cancer, and its overexpression and epigenetic changes could serve as subtype classification biomarkers.

## 1. Introduction

Breast cancer is the most common cancer in women worldwide [1,2]. In recent years, the number of breast cancer patients has risen steadily [3], and there has been a gradual increase in young breast cancer patients [4,5]. Recurrence is very common in breast cancer, and the pattern of recurrence differs depending on the cancer subtype. Nearly 30% of patients experience recurrence in the form of metastasis during follow-up [6]; moreover, these recurrences arise at a steady rate for at least another 15 years after the 5 year treatment period ends [7]. Breast cancer is largely categorized into the luminal, HER2+, and triple-negative breast cancer subtypes based on their immunohistochemical expression pattern of estrogen receptors (ER), progesterone receptors (PR), and human epidermal growth factor receptors (HER2) [8,9]. This hormone receptor-based subtype classification is currently used to target therapy and determine prognosis. However, unpredictability caused by breast cancer heterogeneity limits this approach [10,11]. This warrants efforts to discover more effective and compatible biomarkers that could also serve as therapeutic targets.

Melanoma-associated antigen (MAGE) was originally identified as a melanoma tumor antigen [12] and was developed as an immunotherapy target [13,14]. Recently, it was reported to be linked to tumorigenesis in multiple cancer types [15]. The human superfamily of MAGE proteins is divided into two groups based on their gene expression patterns and the functions of the encoded proteins, namely, type I-cancer/testis antigen MAGEs and type II-ubiquitous MAGEs. The type I MAGE proteins are further subdivided into the MAGE-A, -B, and -C families [16]. The MAGE-A family contains 15 members. In general, they are not expressed in normal tissues due to epigenetic inhibition via DNA hypermethylation, which inactivates the histones at the promoter loci of these genes. In cancer cells, however, this epigenetic regulation undergoes reversible changes that increase the expression of the MAGE-A family genes, thereby promoting cancer progression [17]. In addition, a recent study demonstrated that MAGEA11-overexpressing tumor cells exhibit increased RNA PolII activity and were enriched for the activating histone lysine methylation markers H3K4me3 and H3K79me2 [18]. These findings warrant further studies on the contribution of MAGE-A gene expression and histone modifications at MAGE-A genetic loci to tumor development and the aggressiveness of cancer cells.

Indeed, it was reported that different members of the MAGE-A gene family are upregulated in various types of cancers [19,20,21,22,23,24,25,26,27,28]. For example, MAGEA1 is upregulated in lung cancer, melanoma, and gastric cancer; MAGEA3 is upregulated in breast and lung cancer; MAGEA4 is upregulated in melanoma; MAGEA5 is upregulated in head and neck cancer; and MAGEA9 is upregulated in liver and colon cancer. Interestingly, the expression of various MAGE-A gene family members can correlate strongly. This may reflect their close location on the X chromosome [29,30]. In particular, MAGEA3 and MAGEA6 expression are highly correlated in colon and lung cancer [31], as is MAGEA6 and MAGEA11 expression in prostate cancer [32]. However, it is not clear whether, and which, MAGE-A members are simultaneously expressed in breast cancer.

Several MAGE-A family genes are also associated with poor prognosis. Thus, MAGEA3, MAGEA6, and MAGEA4 expression correlates significantly with lymph node metastasis in oral squamous cell carcinoma [33]. In addition, high expression of MAGEA9 is associated with poor survival in non-small-cell lung carcinoma [34] and hepatocellular carcinoma [35]. Moreover, overexpression of MAGEA2 is associated with an unfavorable prognosis in glioma [36], and MAGEA11 expression correlates positively with poor prognosis in breast cancer [37]. Furthermore, a meta-analysis has shown that high expression of MAGE-A genes is associated closely with poor prognosis in lung, gastrointestinal, breast, and ovarian cancer [38]. These outcomes suggest that MAGE-A is associated with cellular malignancy. This is supported by the fact that MAGE-A proteins function in cell proliferation, cell death, and metastasis through various molecular mechanisms. For example, the overexpression of MAGEA4 prevents apoptosis by reducing cleaved caspase-3 activity [39]. Moreover, in multiple myeloma, MAGEA3 knockdown increases the levels of the proapoptotic proteins BAX and BIM and the cyclin-dependent kinase (CDK) inhibitor p21; this suggests that MAGEA3 represses apoptosis by regulating BAX, BIM, and p21, and induces cell proliferation [40,41]. MAGEA12 also induces the ubiquitination/degradation of p21, thereby promoting cell-cycle progression and apoptosis [42]. However, the roles that MAGE-A gene products play in breast cancer have not yet been established.

To date, the heterogeneity of breast cancer cells has significantly hampered the development of an optimal treatment for all types of breast cancer. New biomarkers are needed to help predict responsiveness to treatment regardless of which hormone receptors are present. In this study, we assessed whether differential MAGE gene expression could be used to generate a new classification of breast cancer subtypes and whether MAGE family members could serve as new therapeutic targets for breast cancer.

## 2. Materials and Methods

### 2.1. Cell Lines and Cell Culture

The human breast cancer cell lines SKBR3, MCF7, and MDAMB231 were purchased from Korean Cell Line Bank (Seoul, Korea). MDAMB468 was obtained from Sapporo Medical University and tested DNA fingerprinting analysis using STR (short-tandem repeat) markers (Korean Cell Line Bank). MDAMB468, SKBR3, and MCF7 cells were maintained as monolayer cultures in Dulbecco’s modified eagle medium (DMEM) supplemented with 10% fetal bovine serum (FBS) and 1% penicillin-streptomycin. MDAMB231 cells were maintained as monolayer cultures in Roswell Park Memorial Institute (RPMI)-1640 medium supplemented with 10% FBS and 1% penicillin-streptomycin. All cells were grown at 37 °C in a humidified atmosphere containing 5% CO_2_.

### 2.2. Small Interfering RNA (siRNA) Transfection

Control siRNAs (sc-37007 and SN-1002) were purchased from Santa Cruz Biotechnology, Inc. (Dallas, TX, USA) and Bioneer (Daejeon, Korea), respectively. For siRNA specific to MAGEA12, predesigned and validated MAGEA12 siRNA (sc-108017) was purchased from Santa Cruz Biotechnology, Inc. (Dallas, TX, USA). Customized MAGEA12 siRNA designed with Invivogen design tool (https://www.invivogen.com/sirnawizard/, accessed 21 May 2021) was synthesized by Bioneer (Daejeon, Korea). The sense and antisense sequences for customized MAGEA12 siRNAs are as follows: 5′-GCUUCCAAGUAGCACUCAGUA-3′ and 5′-UACUGAGUGCUACUUGGAAGC-3′. Cells were seeded and incubated until they reached 60–80% confluence (12–18 h). For transfections, lipofectamine RNAiMAX transfection reagent (Invitrogen, Waltham, MA, USA, 13778150) was diluted in Opti-MEM I reduced serum medium (Gibco, Waltham, MA, USA, 31985-070) and gently mixed with siRNA (10 μM) diluted to a final concentration of 30 nM in the same medium. After a 5 min incubation at room temperature (RT), the resulting siRNA-lipid complexes were added to the cells and incubated for 1–3 days at 37 °C.

### 2.3. Virus Infection for the Stable Cell Line

Cells were seeded in six-well plates and incubated until they reached 50–70% confluence. For infections, the virus (empty vector and MAGEA12-overexpressed) was 1:1 diluted in culture media and added 1 ug/mL polybrene (Sigma-Aldrich, Burlington, VT, USA, TR-1003-G). Next, 24 h later, cells were detached and reseeded in new plates with a final concentration of 2 ug/mL puromycin (Sigma-Aldrich, P9620). Puromycin selection was continued to enrich the population of 80–90%. At this point, cells were diluted and maintained with puromycin.

### 2.4. Quantitative Reverse-Transcription-Polymerase Chain Reaction (qRT-PCR)

Total RNA was isolated from cells using a NucleoSpin RNA/protein kit (MACHEREY-NAGEL, Düren, Germany, 740933.250). cDNA was synthesized from total RNA (1 μg) using M-MLV reverse transcriptase (Promega, Madison, WI, USA, M1705) in a reaction containing RNase inhibitor (Promega, N2111), 100 nM oligo-dT, and 2.5 nM dNTP mixture (Promega, U1205, U1215, U1225, and U1235). qRT-PCR was performed on a LightCycler 96 System (Roche, Basel, Switzerland) using qPCRBIO SyGreen Blue Mix (PCR Biosystems, London, UK, PB20.15) according to the manufacturer’s instructions. The following targets were amplified using the indicated primer pairs (5′-3′): human MAGEA12, GCA GGT CCC CGG CAG TGA T (forward) and AGG GGT GGG TAG GAA ATG TGA GGT (reverse); human MAGEA3, GCA GGT CCC CGG CAG TGA T (forward) and AGG GGT GGG TAG GAA ATG TGA GG (reverse); human MAGEA6, CCC AAG AAG CTG CTC ACC CAA TA (forward) and GGA CCC CAC AGG AAC TCA TAG CAT (reverse); human FA2H, AGG GCG GGC CAG GAC ATC (forward) and CTC AAG GGC TAC AGG CTC GTT CTC (reverse); human ALPP, CCC GCG GCT TCT TCC TCT TC (forward) and CTG GCC CGC CCT CTC AAT G (reverse); human C2orf48, CTG CGC CTC CAC TAT GCT CTC C (forward) and GGC GGC GTG TTC TCC TTG TC (reverse); human DSC2, AAG GAT TGG CGG TGG AGG AGT A (forward) and AAG CCC CAC AGA CCA GCG TAA A (reverse); human FBLN1, GAA CCT GCG GGA CTC TTT TGA CA (forward) and ACG CCC CCG ACC ACA TAG TTC (reverse); human KCNC4, GAG GCC GGC GAC GAT GAG (forward) and CGG CCC GGG AGG AGT AGG (reverse); human EPHA2, CCC CTC CGC CCC ACA CTA C (forward) and GCC CGC ATT CCC CAG ACT C (reverse); human EFNA1, AGG TGA CTG TCA GTG GCA AA (forward) and AGC ACT GTG ACC GAT GCT AT (reverse); human ITGAV, AGG GAT TTT GTC AAG GAG GAT TCA (forward) and TGC TGT AAA CAT TGG GGT CGT ATT (reverse); human RAB1A, GGA GCC CAT GGC ATC ATA GT (forward) and TGG TCA GAT CAC ATT TGT TCC CT (reverse); human FUT8, TGC TAC TGG TGG ATG GGA GA (forward) and GGG AAG CTC GAC CAC TTG AA (reverse); human JAK1, ATG CAC CGG AAA AGC GAT GTC (forward) and ACG GGC CAG GAG GAG GTT TT (reverse); human RPS6KB1, GAG CTG GAG GAG GGG G (forward) and TTT TCT GGC CCT CTG TTC AC (reverse); human S100A8, GAC CTG AAG GTT CTG TTT TTC AGG (forward) and ACT TGT GGT AGA CGT CGA TGA T (reverse); human S100A9, GCT GGA ACG CAA CAT AGA GAC (forward) and CCA GCT CTT TGA ATT CCC CCT (reverse); human CXCL16, CAC GAG GTT CCA GCT CCT TT (forward) and CCA CAA TCC CCG AGT AAG CA (reverse); and human 18s rRNA, CGG CGT CCC CCA ACT TCT (forward) and CGT GCA GCC CCG GAC ATC TA (reverse).

### 2.5. Western Blot Analysis

Protein was extracted using an RNA/protein extraction kit (MACHEREY-NAGEL) according to the manufacturer’s instructions. The proteins in lysates were resolved by sodium dodecyl sulfate-polyacrylamide gel electrophoresis (SDS-PAGE) on 8–10% gels; after which, proteins were transferred to polyvinylidene fluoride (PVDF) membranes. After blocking in phosphate-buffered saline (PBS) containing 1% skim milk and 1.0% Tween-20 (PBST), the membranes were incubated overnight at 4 °C with primary antibodies against MAGE-A (Santa Cruz, sc-20034) and β-actin (Bethyl Laboratories, Montgomery, TX, USA, A300-491A) that were diluted in PBST. The membranes were then washed with PBST and incubated with horseradish peroxidase-conjugated secondary antibodies (diluted in PBST) at RT for 1 h. Immunoreactive proteins were visualized using chemiluminescent reagents (ATTO, Taito, Tokyo, Japan) and detected using an Amersham Imager 600 (GE Healthcare, Chicago, IL, USA).

### 2.6. Proliferation Assay

For proliferation assays of MDAMB231 and MCF7 cells, 5 × 10^4^ cells were resuspended in 2 mL of growth medium and seeded in six-well plates. After 72 h of incubation, the number of cells in each well was counted every 2 days using the trypan blue exclusion method. The viability of MDAMB468 and SKBR3 cells was determined by first, respectively, resuspending 4 × 10^4^ and 5 × 10^4^ cells in 100 μL of growth medium and then seeding the cells in 96-well plates. The cells were grown for the specified period. The luminescence signals were measured using the CellTiter-Glo reagent (Promega, G9241) as per the manufacturer’s protocol.

### 2.7. Migration and Invasion Assay

Transwell migration and invasion assays were performed on 24-well plates containing 8 μm pore polycarbonate membrane inserts (Falcon, Corning, NY, USA, 353097). For invasion assays, the inserts were coated with diluted Matrigel (Corning, Corning, NY, USA, 354230). For all transwell assays, cell suspensions were seeded in the upper chamber and incubated for 1–2 days; after which, the inserts were fixed and stained with crystal violet.

### 2.8. 3D Culture

The three-dimensional (3D) culture method has been described previously [43]. In brief, after thawing basement membrane extracts (BME; Cultrex, 3445-005-01) at 4 °C overnight, 80 μL of BME was added to each well of a 48-well plate, and the plates were incubated at 37 °C for 30 min. MDAMB468 and MCF7 cells were seeded at 2.1 × 10^4^ cells/cm^2^ and 3.1 × 10^4^ cells/cm^2^, respectively, in 100 μL of H14 medium (DMEM/F12) containing 5 μg/mL prolactin, 250 ng/mL insulin, 1.4 μM hydrocortisone, 0.1 nM β-estradiol, 2.6 ng/mL sodium selenite, 10 μg/mL transferrin, and 5 ng/mL epidermal growth factor. The cells were incubated at 37 °C for 5 min; after which, 10% BME in H14 medium was added. The 3D cultures were maintained for 4 days. The H14 medium was changed every 2 days.

### 2.9. ChIP-qPCR

Chromatin immunoprecipitation (ChIP) assays were performed as described previously [44,45]. In brief, 5 × 10^6^ cells were treated with 1% formaldehyde (Sigma, F8775) at RT for 10 min to crosslink DNA, and the reaction was stopped by adding 0.125 M glycine and incubating the mixture at RT for 10 min. The cells were then pelleted by centrifugation, lysed, and subjected to 10 cycles of sonication; after which, sonicated chromatin was immunoprecipitated with 1 μg of antibody and 10 μL of Protein A beads. Crosslinks in the eluate from washed immunocomplexes were reversed by incubating with proteinase K; after which, the immunoprecipitated DNA was purified with a QIAquick PCR purification kit (QIAGEN, Hilden, Germany, 28106) using 50 μL of elution buffer. qRT-PCR was performed on the diluted ChIP DNA using SYBR Green mix and the following primer pairs (5′-3′): human negative control region, TCC TAT TCA AGT CCT TCC TCC A (forward) and TGC AAA ACA TAT GAA ACA CAA GC (reverse); human EFNA1 promoter region, GGG ACA GGA AGC CAT GAG TA (forward), and GGA GGT GGG TAA GGA AGA GG (reverse).

### 2.10. RNA-Seq Library

Total RNA was extracted using a Direct-zol RNA prep kit (Zymo, Irvine, CA, USA, R2071), and RNA-seq libraries were constructed using the NEXTflex rapid directional mRNA-seq kit (PerkinElmer, Waltham, MA, USA, NOVA-5138-11). In brief, 5–10 μg of purified RNA was poly-A–selected and fragmented with fragmentation enzyme. Following first- and second-strand synthesis from a template of poly-A–selected fragmented RNA, other procedures, from adenylation to PCR amplification, were performed according to the RNA-seq library construction steps provided by the manufacturer.

### 2.11. ChIP-Seq Library Construction

ChIP-seq libraries were constructed using the NEXTflex ChIP-seq kit (PerkinElmer, NOVA-5143-02) according to the manufacturer’s instructions. In brief, 40 μL of purified ChIP DNA was end-repaired and size-selected (250–300 bp) using AMPure XP beads. Other procedures, from adenylation to PCR amplification, were performed according to the ChIP-seq library construction steps provided by the manufacturer. The quality of the ChIP-seq library was assessed with a bioanalyzer using a high-sensitivity chip (Agilent), which confirmed that the average size of ChIP-seq libraries ranged from 250 to 350 bp. For multiplexing, equal molar quantities of libraries were combined by considering sequencing depth per sample (20–40 million reads per library). ChIP-seq libraries were sequenced using an Illumina NextSeq platform with single-end reads of 76 bases.

### 2.12. Data Analysis

All raw and analyzed data were accessed in gene expression omnibus (GEO). The accession numbers are series GSE85158 (samples GSM2258722, GSM2258731, GSM2258732, GSM2258794, GSM2258802, GSM2258805, GSM2258848, GSM2258856, GSM2258858, GSM2258884, GSM2258892, and GSM2258894); and series GSE96860 (samples GSM2545229, GSM2545230, GSM2545231, GSM2545232, GSM2545245, GSM2545246, GSM2545247, GSM2545248, GSM2545257, GSM2545258, GSM2545259, GSM2545260, GSM2545265, GSM2545266, GSM2545267, and GSM2545268). The RNA-seq source data of 70 breast cancer cell lines were obtained from specific reference [46]. Data quality was checked using FastQC, and adapter sequences were removed using Cutadapt. Up to this point, ChIP-seq and RNA-seq followed the same process. Thereafter, the ChIP-seq data were mapped to the reference genome using Bowtie2 and normalized tag count and visualized using HOMER. By contrast, the RNA-seq data were mapped to the reference using STAR, and bam files were converted to tdf files using igv tools. The data were loaded and viewed using integrative genomics viewer (IGV) (version 2.4.13).

### 2.13. Statistical Analysis

Statistical analyses were performed by using two-tailed paired *t*-test. All analyses were conducted with GraphPad Prism 5.01 Software (GraphPad, San Diego, CA, USA). All data were presented as mean ± SD. *p*-values < 0.05 indicated statistically significant differences. *p*-values indicated by *, **, and *** signify < 0.01, < 0.001, and < 0.0001, respectively.

## 3. Results

### 3.1. A Subset of Breast Cancer Cells Expresses Both MAGEA12 and MAGEA3 in a Subtype-Independent Manner

**Figure 1:**
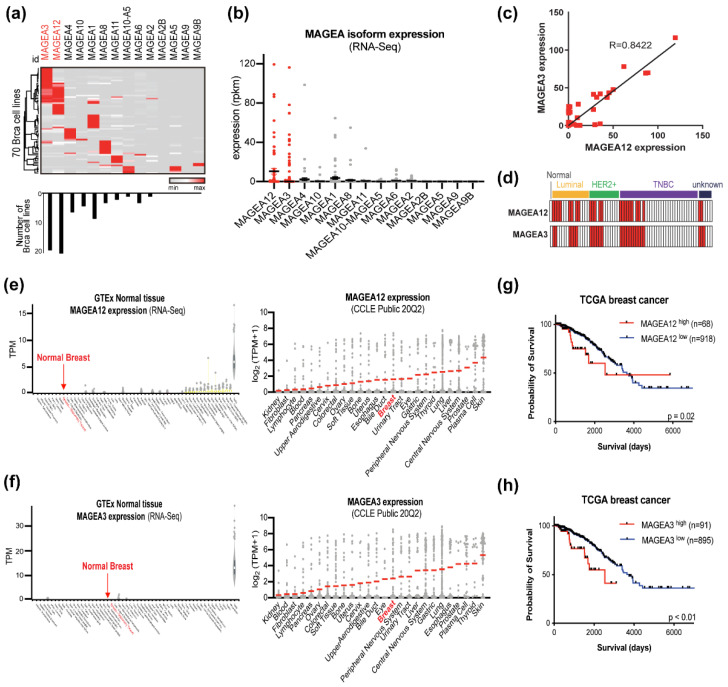
Expression of MAGE-A family genes in breast cancer cell lines. (**a**) A heat map (**top**) shows the expression pattern of the MAGE-A isoforms in 70 breast cancer cell lines. A plot (**bottom**) shows the numbers of breast cancer cell lines that expressed each MAGE-A isoform (defined as rpkm > 5 MAGE-A expression). Thus, there were 18, 19, 8, and 6 cell lines that expressed MAGEA3, MAGEA12, MAGEA1, and MAGEA4, respectively. (**b**) MAGE-A isoform expression levels in 70 breast cancer cell lines, as determined from RNA-seq data. MAGEA12 had the highest median expression value. (**c**) Correlation between MAGEA12 and MAGEA3 expression in the 70 cell lines (R = 0.8422). (**d**) Relationship between the breast cancer subtypes of the cell lines and their expression of MAGEA12 and/or MAGEA3. No relationship was observed. (**e**) Expression levels of MAGEA12 in normal tissues (GTEx) and cancer cells (CCLE). (**f**) Expression levels of MAGEA3 in normal tissues (GTEx) and cancer cells (CCLE). (**g**,**h**) Kaplan-Meier analysis of the overall survival of 986 breast cancer patients who were stratified into two groups depending on whether MAGEA12 (**g**) and MAGEA3 (**h**) expression was ‘high’ (red) and ‘low’ (blue). The stratification was conducted with an autoselected best cutoff.

To investigate the expression patterns of the MAGE-A isoforms in breast cancer, we analyzed the publicly available RNA-seq data of 70 breast cancer cell lines (Figure 1a and Table S1) [46]. This analysis confirmed that in general, each cell line expressed a single MAGE-A isoform, with the most common isoform being MAGEA12. However, a subset of breast cancer cell lines, including MDAMB468 and SKBR3, expressed both MAGEA12 and MAGEA3. Overall, 19 breast cancer cell lines expressed MAGEA12, and 13 of these also expressed MAGEA3. The RNA-seq data also indicated that the most highly expressed MAGE-A isoform in breast cancer cell lines was MAGEA12, followed by MAGEA3 (Figure 1b). We also showed that the expression levels of these two genes in breast cancer cells correlated strongly (Figure 1c). This suggests that the expression of MAGEA12 and MAGEA3 is co-regulated. Significantly, the four breast cancer subtypes did not differ in terms of MAGEA12 and MAGEA3 expression patterns (Figure 1d). Next, we examined the expression of MAGEA12 and MAGEA3 in normal tissues and various cancer cell lines by using genotype-tissue expression (GTEx) and Cancer Cell Line Encyclopedia (CCLE), respectively. While both MAGEA12 and MAGEA3 were weakly expressed in normal tissues, including the breast, they were generally upregulated to varying degrees in multiple cancers (Figure 1e,f). We also conducted an overall survival analysis of 986 breast cancer patients in the TCGA database whose MAGEA12 and MAGEA3 expression in their tumors had been measured. When the patients were stratified according to whether MAGEA12 or MAGEA3 expression was high or low, the patients with high expression of these genes had a significantly worse prognosis (Figure 1g,h). Thus, our data suggest that the aberrant expression of MAGEA12 and MAGEA3 genes may be useful for classifying and predicting malignant breast cancer phenotypes.

### 3.2. Chromatin Modifications at MAGE-A Gene Loci in Breast Cancer Cell Lines

**Figure 2:**
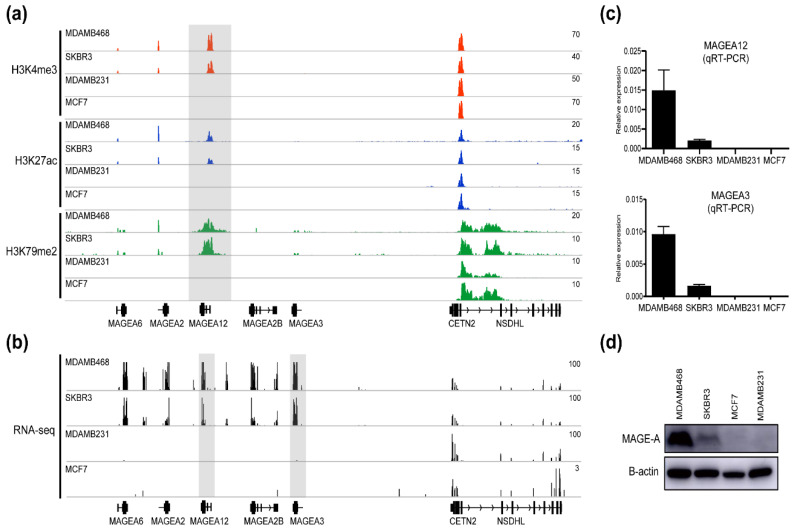
Histone modifications at the MAGEA12 locus in four breast cancer cell lines that have high or low MAGEA12 and MAGEA3 expression. (**a**) IGV genome browser snapshot showing the ChIP-seq data for H3K4me3, H3K27ac, and H3K79me2 in the MAGEA12 high-expressing (MDAMB468; TNBC and SKBR3; HER2+) and low-expressing (MDAMB231; TNBC and MCF7; Luminal) cell lines. (**b**) Snapshot of RNA-seq reads at the MAGEA12-associated loci in the four cell lines. (**c**) qRT-PCR expression of MAGEA12 and MAGEA3 in the four cell lines. (**d**) Western blot analysis of MAGE-A proteins in the four cell lines.

To determine whether the altered expression of the MAGE-A isoforms in breast cancer cells is due to epigenetic changes, we used chromatin immunoprecipitation sequencing (ChIP-seq) to assess the changes in the histone modifications H3K4me3, H3K27ac, and H3K79me2 in different breast cancer cell lines. These histone modifications correspond to actively transcribed genomic regions. We focused on four cell lines that expressed high (MDAMB468; TNBC and SKBR3; HER2+) or low (MDAMB231; TNBC and MCF7; Luminal) levels of both MAGEA12 and MAGEA3. We found massive enrichment of H3K4me3, H3K27ac, and H3K79me2 at the MAGEA12 locus in the MDAMB468 and SKBR3 cell lines compared to the MDAMB231 and MCF7 cell lines (Figure 2a). By contrast, the CETN2 locus close to the MAGEA loci did not accumulate these histone modifications in any of the four cell lines. To determine whether these differences in chromatin modification at the MAGEA12 locus were associated with different levels of MAGEA12 gene expression, we assessed the RNA-seq data relating to the MAGEA12 locus by using the IGV genome browser (Figure 2b). Tag enrichment obtained through RNA-seq was clearly capable of distinguishing between the cell lines that expressed high or low MAGEA12, which is consistent with the accumulation patterns of the histone markers. Quantitative RT-PCR (qRT-PCR) analysis then confirmed that the mRNA expression of MAGEA12 and MAGEA3 was higher in the MDAMB468 cells and, to a lesser extent, in SKBR3 cells, than in the MDAMB231 and MCF7 cells (Figure 2c). Western blotting analysis using an antibody that recognizes both MAGEA12 and MAGEA3 further supported the qRT-PCR results (Figure 2d). These results indicate that MAGEA12 and potentially MAGEA3 are upregulated at the transcriptional level via epigenetic changes in a subset of breast cancer cell lines.

### 3.3. Identification of MAGEA12 Signature Genes in Breast Cancer

**Figure 3:**
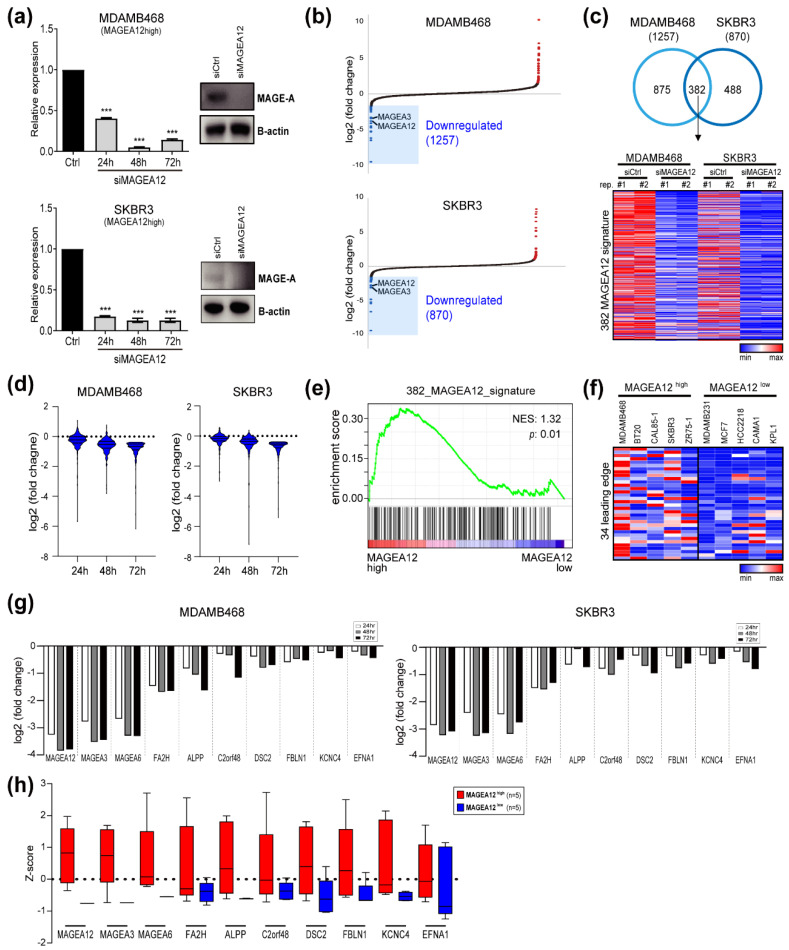
Identification of MAGEA12-regulated genes in breast cancer cells. (**a**) Expression of MAGEA12 in MDAMB468 and SKBR3 cells 24, 48, and 72 h after treatment with siRNA against MAGEA12, as determined by qRT-PCR. Western blotting was also conducted 48 h after treatment (*** *p*-value < 0.0001). (**b**) Waterfall plots showing the differentially expressed genes (DEGs) in the MAGEA12-knockdown cells. DEGs were defined as the genes that had >1.3-fold lower expression in the MAGEA12-knockdown cells compared to in control cells. (**c**) Venn diagram showing the genes that were downregulated by siRNA against MAGEA12 in MDAMB468 and SKBR3 cells. The 382 signature genes that were downregulated in both cells are shown in the heat map below. (**d**) Expression of the 382 signature genes over time after treatment with siMAGEA12. (**e**) GSEA of the 382 signature genes in 4 breast cancer cell lines with high or low MAGEA12 expression. (**f**) Heat map showing the expression of the 34 leading-edge genes from the GSEA analysis in the 10 breast cancer cell lines with high or low MAGEA12 expression. Leading-edge genes indicated the subset of genes that contributed the most to the enrichment signal (ES) in the GSEA analysis. (**g**) Effect of MAGEA12-knockdown on the expression of the selected of the 34 leading-edge genes in the MDAMB468 and SKBR3 cells. (**h**) Expression of selected leading-edge genes in breast cancer cell lines that had high or low MAGEA12 expression.

To explore the function of MAGEA12 in breast cancer, we used small interfering RNA (siRNA) specific for this gene (siMAGEA12) to suppress the endogenous MAGEA12 expression in the cell lines that expressed MAGEA12 at high levels (i.e., MDAMB468 and SKBR3) and then subjected these cells to RNA-seq (Figure 3 and Table S2). First, we assessed the effect of the siRNA treatment on the expression of MAGEA12 at 24, 48, and 72 h (Figure 3a). In MDAMB468 cells, MAGEA12 expression decreased by more than 60% at each time point: at 48 and 72 h, expression was reduced by ~80–90%. In SKBR3 cells, MAGEA12 expression was reduced by ~90% at all time points. The siMAGEA12 treatment had similar effects on MAGEA12 protein levels at 72 h time point. RNA-seq then revealed which genes were downregulated in MDAMB468 and SKBR3 cells 72 h after siMAGEA12 treatment (Figure 3b). In total, 1257 and 870 genes were downregulated in MDAMB468 and SKBR3, respectively; of these, 382 were downregulated in both cell lines and thus constituted the MAGEA12 signature genes (Figure 3c). The expression of all 382 MAGEA12 signature genes gradually decreased in a time-dependent manner after siMAGEA12 treatment (Figure 3d). This indicates that these genes are primary downstream targets of MAGEA12.

Next, to determine the functional relevance of these signature genes, we conducted a gene set enrichment analysis (GSEA) on five MAGEA12-high and five MAGEA12-low cell lines. This showed significant enrichment of the 382 signature genes in the cell lines with high MAGEA12 expression and low expression of these genes in the MAGEA12-low cell lines (Figure 3e). GSEA further indicated that 34 leading-edge genes were among the genes with high enrichment scores. In addition, this analysis confirmed that the expression of these 34 leading-edge genes was increased in most of the MAGEA12-high cell lines, unlike the MAGEA12-low cell lines (Figure 3f). However, in one MAGEA12-high (ZR75-1), the expression of the 34 leading-edge genes was similar to that in the MAGEA12-low cell lines. Thus, in a subset of breast cancer cell lines, the 34 leading-edge genes may be regulated in a MAGEA12-independent manner. The qRT-PCR analysis of MAGEA12-depleted MDAMB468 and SKBR3 cells validated the MAGEA12-dependent expression of the aforementioned leading-edge genes, including FA2H, ALPP, C2orf48, DSC2, FBLN1, KCNC4, and EFNA1 (Figure 3g). We also found that the expression of MAGEA3 and MAGEA6 was reduced by MAGEA12 knockdown, suggesting the master regulatory role of MAGEA12. These genes, and other leading-edge genes, were also generally expressed at much lower levels in the MAGEA12-low cell lines than in the MAGEA12-high cell lines (Figure 3h). To confirm that the above results are an on-target effect of MAGEA12 siRNA, we synthesized custom siRNA and successfully reproduced the results in Figure 3g,h (Figure S1). Taken together, these results suggest that 382 MAGEA12-regulated signature genes participate in breast cancer.

### 3.4. Effect of MAGEA12 Silencing and Overexpression on Breast Cancer Cell Aggressiveness

**Figure 4:**
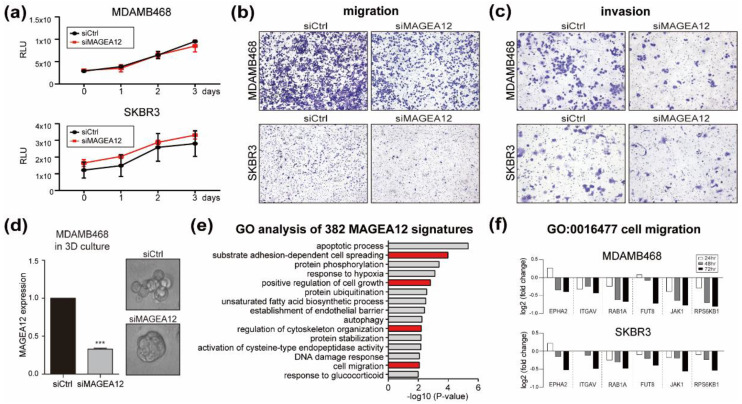
MAGEA12 knockdown suppresses the aggressiveness of breast cancer cells. (**a**) Viability of MDAMB468 and SKBR3 cells after siMAGEA12 treatment. (**b**) Migration and (**c**) invasion of MAGEA12-knockdown MDAMB468 and SKBR3 cells. (**d**) qRT-PCR analysis of siMAGEA12-treated 3D-cultured MDAMB468 cells (**left**) and images of the 3D cultures (**right**). (*** *p*-value < 0.0001). (**e**) GO enrichment analysis of the biological process terms that were associated most prominently with the 382 signature genes. (**f**) Expression of the GO:0016477 term (cell migration)-annotated genes in MDAMB468 and SKBR3 cells at each time point after MAGEA12-silencing treatment.

We then asked whether the putative MAGEA12-regulated genes contributed to the characteristics of breast cancer cells by analyzing the proliferation, invasion, and migration of the MAGEA12-knockdown breast cancer cells (Figure 4). Downregulation of MAGEA12 did not affect the viability of MDAMB468 or SKBR3 cells (Figure 4a). However, suppressing MAGEA12 expression decreased the migratory and invasive capacities of both cell lines, notwithstanding the differences between the lines in terms of migration and invasion (Figure 4b,c). In addition, we showed that MAGEA12 silencing reduced breast cancer aggressiveness by using a 3D culture system that can identify aggressive characteristics based on a cell shape classification [32,33,34]. Thus, siRNA-mediated MAGEA12 knockdown not only dramatically reduced the expression of MAGEA12, but it also changed the morphology of the cell clusters from their usual aggressive grape-like phenotype to the less aggressive rounded/mass cluster phenotype (Figure 4d).

This suggests that MAGEA12 affects cell–cell adhesion and thus may be involved in the aggressiveness of breast cancer cells. These phenotypic results are consistent with a gene ontology (GO) analysis of the MAGEA12 signature genes, which showed that the most prominent terms that were associated with these genes were substrate adhesion-dependent cell spreading, regulation of cytoskeleton organization, and cell migration (Figure 4e). Notably, the expression of the cell-migration-associated genes of MDAMB468 and SKBR3 decreased in a time-dependent manner after siMAGEA12 treatment (Figure 4f). These results suggest that MAGEA12 may help regulate the aggressiveness of breast cancer cells.

**Figure 5:**
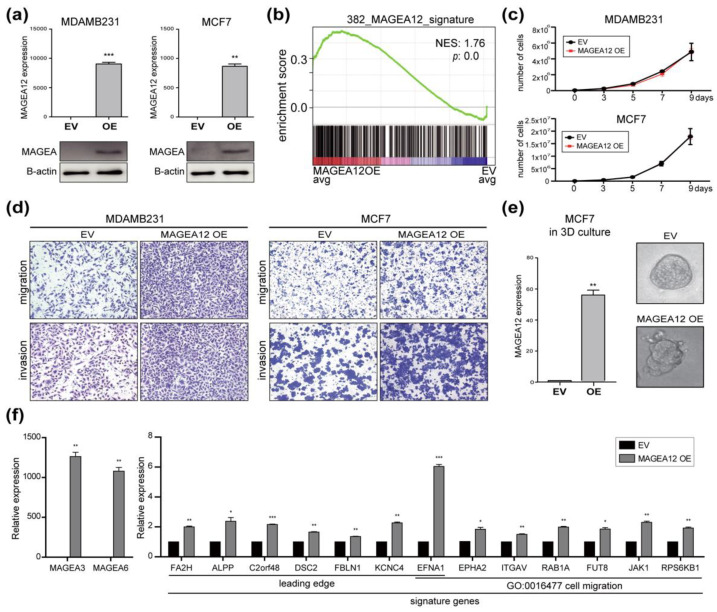
MAGEA12 overexpression promotes aggressive behavior in breast cancer cells. (**a**) MAGEA12 levels in MAGEA12-overexpressing MDAMB231 and MCF7 cells, as determined by qRT-PCR (**top**) and Western blotting (**bottom**). (** *p*-value < 0.001, *** *p*-value < 0.0001). (**b**) GSEA of the 382 signature genes in breast cancer cell lines relative to their MAGEA12 expression. (**c**) Proliferation of the MAGEA12-overexpressing cell lines between 0 and 9 days. (**d**) Migration and invasion assays with the MAGEA12-overexpressing cell lines. (**e**) 3D culture of MCF7 cells that overexpressed MAGEA12 or empty vector (control). (** *p*-value < 0.001). (**f**) Expression of MAGEA3, MAGEA6, selected target genes in MAGEA12-overexpressing cell. (* *p*-value < 0.01, ** *p*-value < 0.001, *** *p*-value < 0.0001).

To confirm that MAGEA12 functions relate to breast cancer aggressiveness, we induced MDAMB231 and MCF7 cells, which do not express MAGEA12, to stably overexpress MAGEA12 (Figure 5). qRT-PCR and Western blot analyses indicated that these cells showed a marked increase in MAGEA12 expression (Figure 5a). Interestingly, GSEA of the 382 MAGEA12 signature genes in the MAGEA12-overexpressing cells showed that the relative expression of MAGEA12 increased significantly (Figure 5b), which further verifies that the expression of the 382 signature genes is regulated by MAGEA12 expression. As expected, the overexpression of MAGEA12 did not increase the proliferation of MDAMB231 or MCF7 cells (Figure 5c) but did enhance their migratory and invasive abilities (Figure 5d). We also showed that the MAGEA12-overexpressing MCF7 cell line expressed high levels of MAGEA12 in even 3D cultures, and that this was associated with a change in their morphology. These cells formed grape-like clusters, which is consistent with their potential origin from metastatic tumor cells (Figure 5e). The phenotypic consequences of MAGEA12 overexpression were supported by the upregulation of the leading-edge genes and genes related to cell migration as well as MAGEA3 and MAGEA6 (Figure 5f). Taken together, these results support the notion that increased expression of MAGEA12 may contribute to the aggressiveness of breast cancer cells.

### 3.5. FOXA1 Is a Candidate Transcription Factor That Regulates MAGEA12 Signature Genes

**Figure 6:**
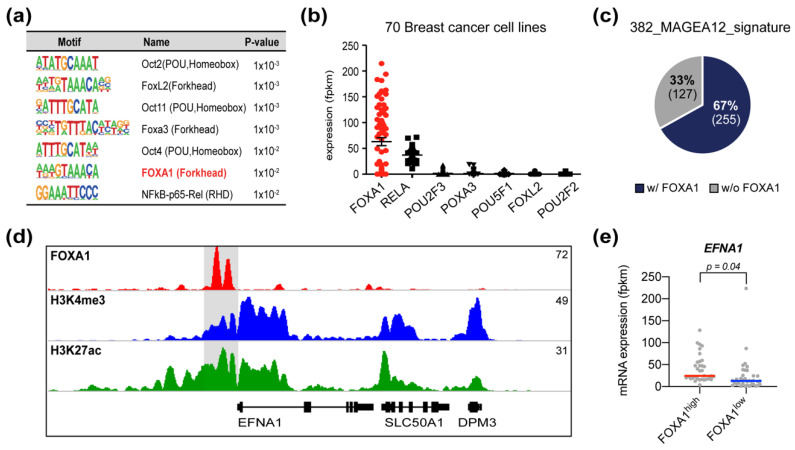
FOXA1 is a candidate transcription factor in the MAGEA12 pathway. (**a**) Motif analysis of the promoter regions of the 382 MAGEA12 signature genes. (**b**) RNA-seq data of 70 breast cancer cell lines showing the expression levels of the candidate transcription factors. (**c**) Pie chart depicting the frequency of the 382 MAGEA12 signature genes with and without FOXA1-binding motifs. (**d**) IGV genome browser snapshot showing FOXA1, H3K4me3, and H3K27ac ChIP-seq data at the EFNA1 locus. (**e**) Expression of EFNA1 in the 70 breast cancer cell lines that were divided into those that expressed FOXA1 at high or low levels.

To determine which transcription factors regulate the expression of the MAGEA12 signature genes, we subjected the promoter regions of the MAGEA12 signature gene loci to motif analysis (Figure 6a). OCT2, FOXL2, OCT11, FOXA3, OCT4, FOXA1, and NF-κB binding motifs were found in the promoters with high significance. To determine whether these transcription factors are expressed in breast cancer cells, we analyzed the RNA-seq data of 70 breast cancer cell lines. This showed that forkhead box A1 (FOXA1) was expressed at much higher levels in these lines than the other candidate transcription factors (Figure 6b). Notably, FOXA1 is expressed specifically in breast cancer cells and is associated with open chromatin and ERα expression [47,48]. We then asked whether FOXA1 regulates the 382 MAGEA12 signature genes by using ChIP-seq for FOXA1. This showed that 255 of the 382 MAGEA12 signature genes contained FOXA1-binding motifs in their promoter region (Figure 6c and Table S3). One of these was the gene for Ephrin A1 (EFNA1), which is involved in adhesion and migration [49] and was one of the 34 leading-edge genes as well as in association with cell migration (Figure 3g, Figure 4f and Figure S1). Notably, our analysis showed that its promoter region contained FOXA1-binding motifs that bore the H3K4me3 and H3K27ac modifications (Figure 6d). In addition, when we divided the 70 breast cancer cell lines into those that had high or low FOXA1 expression, we found that EFNA1 expression was significantly higher in the lines with high FOXA1 expression (Figure 6e). These results suggest that the MAGEA12 signature genes may be regulated by FOXA1.

### 3.6. Chromatin Modifications in MAGEA12 Signature Genes Parallel MAGEA12 Expression Levels

**Figure 7:**
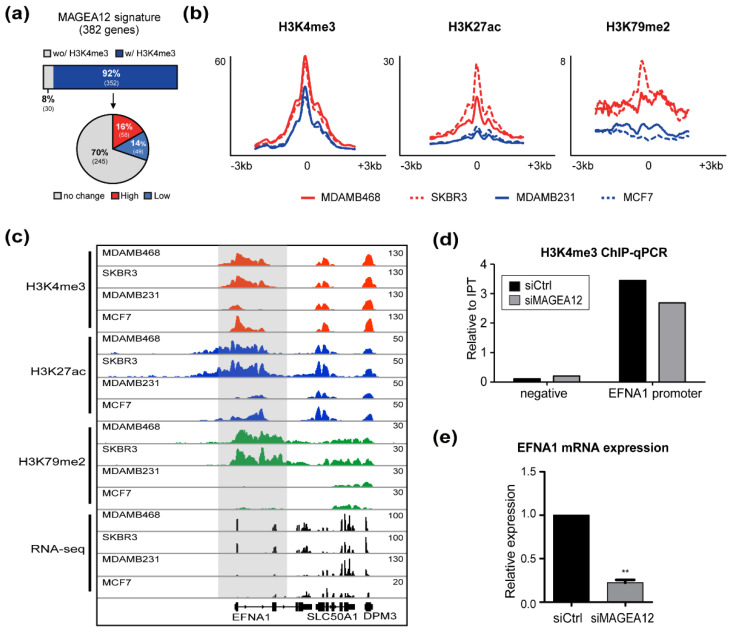
MAGEA12 levels parallel the levels of active histone modification markers in a subset of genes in breast cancer cells. (**a**) The bar graph (**top**) shows the frequency of the 382 MAGEA12 signature genes that did and did not bear the H3K4me3 marker. Of these genes, 352 bore H3K4me3 markers. The pie chart (**bottom**) shows the proportion of these 352 genes whose H3K4me3 levels were elevated in the high MAGEA12-expressing cells and concomitantly reduced in low MAGEA12-expressing cell lines (MDAMB231 and MCF7) (red), decreased in the high MAGEA12-expressing lines and increased in the low MAGEA12-expressing lines (blue), or did not change regardless of MAGEA12 expression (grey). (**b**) Density plots of H3K4me3, H3K27ac, and H3K79me2 in the four cell lines with high (red lines) or low (blue lines) MAGEA12 expression. (**c**) IGV genome browser snapshot showing the H3K4me3, H3K27ac, and H3K79me2 ChIP-seq and RNA-seq data at the aggr gene locus for the four cell lines with high and low MAGEA12 expression. (**d**) H3K4me3 ChIP-qPCR at the EFNA1 promoter compared with that at a negative control region in siMAGEA12-treated MDAMB468 cells. (**e**) Expression of EFNA1 in siMAGEA12-treated MDAMB468 cells, as determined by qRT-PCR. (** *p*-value < 0.001).

We next examined whether the level of MAGEA12 expression affected the chromatin modifications in, and the promoter activity of, the 382 MAGEA12 signature genes by using the H3K4me3 ChIP-seq data of the four cell lines that expressed MAGEA12 at high (MDAMB468 and SKBR3) or low (MDAMB231 and MCF7) levels. This analysis showed that 352 (92%) of the MAGEA12 signature genes were enriched for H3K4me3 in the promoter region (Figure 7a and Table S4). Moreover, 58 (16%) of these showed positive changes in the H3K4me3 pattern that corresponded with the MAGEA12 expression levels. Specifically, the promoter regions of these 58 genes had higher levels of the H3K4me3, H3K27ac, and H3K79me2 markers in the cell lines with high MAGEA12 expression compare to the cell lines with low MAGEA12 expression (Figure 7b), exemplified by EFNA1 (Figure 7c). It had higher levels of the active markers H3K4me3, H3K27ac, and H3K79me2 in the MAGEA12-high breast cancer cell lines (Figure 7c). Notably, a visual representation of RNA-seq data showed a higher density of mapped reads at the EFNA1 gene structure in the high MAGEA12-expressing cell lines compared in the MAGEA12-low cell lines (Figure 7c). Next, to confirm that enrichment of H3K4me3 was affected by MAGEA12 expression levels, we treated the high MAGEA12-expressing MDAMB468 cell line with siMAGEA12 and performed H3K4me3 ChIP-qPCR. This showed that the siMAGEA12-treated cells had lower levels of H3K4me3 in the EFNA1 promoter region relative to the levels in IPT, a negative control region (Figure 7d). To confirm that these chromatin changes affected EFNA1 gene expression, we performed a qRT-PCR analysis (Figure 7e). This showed that EFNA1 expression was reduced by up to 70% in the MAGEA12-knockdown cells compared with the control cells. Furthermore, transcription of some of the other 34 leading-edge genes was also decreased when MAGEA12 expression was downregulated (Figure S2). Taken together, these results suggest that MAGEA12 regulates the expression of its target genes in breast cancer cells by inducing histone modifications.

## 4. Discussion

Hormone receptor and HER2-based breast cancer therapy can be successful, but its efficacy is limited by the heterogeneity and complexity of breast cancer cells. This has led to the idea that an approach that transcends the hormone receptor concept is needed, particularly one that is based on a compatible biomarker of breast cancer cell aggression and resistance. In this study, we provided evidence that suggests MAGEA12 expression and histone alteration of its locus in the genome are aggressiveness-related markers in breast cancer.

Although various cancers demonstrate MAGE-A family gene overexpression and the accumulation of their encoded proteins [19,20,21,22,23,24,25,26,27,28], the specific MAGE-A gene products that are functionally relevant in breast cancer remain largely undetermined. To address this, we first analyzed the RNA-seq data of 70 breast cancer cell lines to determine the breast-cancer-related expression patterns of the MAGE-A gene family members. While some of the cell lines showed little or no expression of any MAGE-A family genes, most expressed one or two of the MAGE-A family genes. MAGEA12 and MAGEA3 were the predominant isoforms that were expressed by breast cancer cells; their expression levels were also high compared with those of other genes belonging to the MAGE-A family. Notably, previous studies have reported that the expression of some MAGE-A family genes is co-regulated in various cancers [31,32]. For example, MAGEA3 and MAGEA6 are both associated with colorectal and lung cancers, and MAGEA6 and MAGEA11 are coexpressed in prostate cancer. Consistent with this, we found that MAGEA12 and MAGEA3 expression was strongly correlated in breast cancer. Significantly, we also observed that breast cancer patients with high MAGEA12 and MAGEA3 expression had a poor prognosis. Therefore, we propose that monitoring the expression of both MAGEA12 and MAGEA3 may help predict the prognosis and malignancy of breast cancer better than the expression of other members of the MAGE-A family genes.

While the expression of MAGE-A family genes is inhibited by DNA hypermethylation in somatic tissues, it has been reported that in cancer, the promoters of these genes become demethylated and their expression increases, thus promoting the growth of the cancer cells. For example, in prostate, ovarian, and colon cancer cells, the MAGEA1 and MAGEA11 promoters are hypomethylated, and their expression is increased [50,51]. However, other studies have also shown that promoter demethylation is not sufficient to increase the expression of the MAGE-A genes. Rather, it has been proposed that MAGE-A gene expression is associated with histone activation, as evidenced by the upregulation of the MAGE-A genes after treatment with a histone deacetylase inhibitor [52]. We confirmed in the present study that the MAGEA12 promoter in cell lines with high, but not low, MAGEA12 expression is enriched for H3K4me3, H3K27ac, and H3K79me2 markers. This suggests that MAGEA12 expression is regulated by chromatin changes. Moreover, the fact that histone modifications at the MAGEA12 locus correlated strongly with MAGEA12 expression levels suggests that both the expression levels of MAGEA12 and the unique characteristics of the histone markers at its locus have potential as biomarkers for classifying breast cancer cells.

It has been reported that MAGE-A gene products are involved in ubiquitination, proliferation, and apoptosis [31,41,53,54,55]. The relationship between MAGE-A proteins and ubiquitination is well known; for example, MAGEA3 and MAGEA6 form an E3 ubiquitin ligase complex with TRIM28, which participates in the survival of cancer cells by degrading AMPKa1 [31,55]. In addition, MAGE-A proteins can bind directly to p53, thereby regulating the targets of p53 and, ultimately, the cell-cycle progression and apoptosis of cancer cells [41,54]. However, the relevant functions of MAGEA12 in breast cancer remain unclear. In the present study, we detected 382 MAGEA12 signature genes through transcriptome analyses and showed that these signature genes are associated with the malignancy and aggressiveness of breast cancer cells. We also found that MAGEA12 levels correlated with changes in breast cancer cell motility and invasion in monolayer cultures as well as the formation of cancer stem cell-like tumorspheres under 3D culture conditions. In addition, promoter analysis of the MAGEA12 signature genes showed that the transcription factor FOXA1 may regulate the expression of many of the MAGEA12 signature genes; thus, we demonstrated that FOXA1 was strongly expressed in breast cancer cell lines and an investigation of FOXA1 binding at the whole-genome level using ChIP-seq showed that FOXA1 occupied the promoters of many MAGEA12 signature genes. It has been reported that FOXA1 cooperates with estrogen receptor-α (ESRa) to regulate chromatin accessibility in breast cancer [56,57,58]. However, no link between FOXA1 and MAGEA12 has yet been reported. Our findings suggest that MAGEA12 may collaborate with FOXA1 to regulate the aggressiveness of breast cancer cells.

Finally, we propose that MAGEA12 plays a role in maintaining chromatin activation. In breast cancer cell lines that had high levels of MAGEA12 expression, the promoters of the MAGEA12 signature genes were enriched for H3K4me3. In particular, we showed that siRNA-mediated MAGEA12 knockdown reduced H3K4me3 levels at the promoter of one of the MAGEA12 signature genes, namely, EFNA1. Given that diminished H3K4me3 levels are associated with reduced expression of EFNA1, we suggest that MAGEA12 acts through an, as of yet, unknown mechanism to activate chromatin and thereby regulate the transcription of genes involved in breast cancer cell malignancy.

## 5. Conclusions

In conclusion, we found that MAGEA12 is associated significantly with aggressiveness in breast cancer regardless of the hormone receptor subtype status. Moreover, we observed that the MAGEA12-regulated signature genes are involved in breast cancer cell migration and invasion and that the regulation of MAGEA12 expression could play an important role in determining the shape of aggressive breast cancer cells. In addition, we showed that MAGEA12 could regulate the expression of signature genes via chromatin modifications. These results suggest that the overexpression of MAGEA12 may contribute to the metastasis of breast cancer cells and that histone modifications that are regulated by MAGEA12 could be potential markers of breast cancer aggressiveness.

## Data Availability

Data are available upon request from the corresponding author.

## Supplementary Materials

The following are available online at https://www.mdpi.com/article/10.3390/cancers13133176/s1, Figure S1: Validation of MAGEA12 siRNA treatment, Figure S2: Confirmation of MAGEA12 target gene expression, Table S1: Expression level of MAGEA family genes in 70 breast cancer cell lines, Table S2: RNA-seq analysis of gene expression in MAGEA12 knock-down cells, Table S3: Enrichment of FOXA1 at MAGEA12-signature gene loci, and Table S4: Enrichment of H3K4me3 at MAGEA12-signature gene loci.
