# Supplementary material for: Epigenetic Upregulation of MAGE-A Isoforms Promotes Breast Cancer Cell Aggressiveness
Source: Cancers (Basel). 2021 Jun 25;13(13):3176. doi: 10.3390/cancers13133176 (PMC8268034; doi:10.3390/cancers13133176)
Supplement: Supplementary file 1 [file cancers-13-03176-s001.zip › cancers-1194237-supplementary/supplementary figures_062421/Supplementary figures_final.pdf]

Supplementary Materials

Epigenetic upregulation of MAGE-A isoforms supports aggressive breast cancer cells

Chaeun Oh [1,†], Hwa-Ryeon Kim [2,†], Sumin Oh [1,3], Je Yeong Ko [4], Yesol Kim [4], Keunsoo Kang [5], Young Yang [4], Jongmin Kim [4], Jong Hoon Park [4], Jae-Seok Roe [2,*] and Kyung Hyun Yoo [1,3,*]

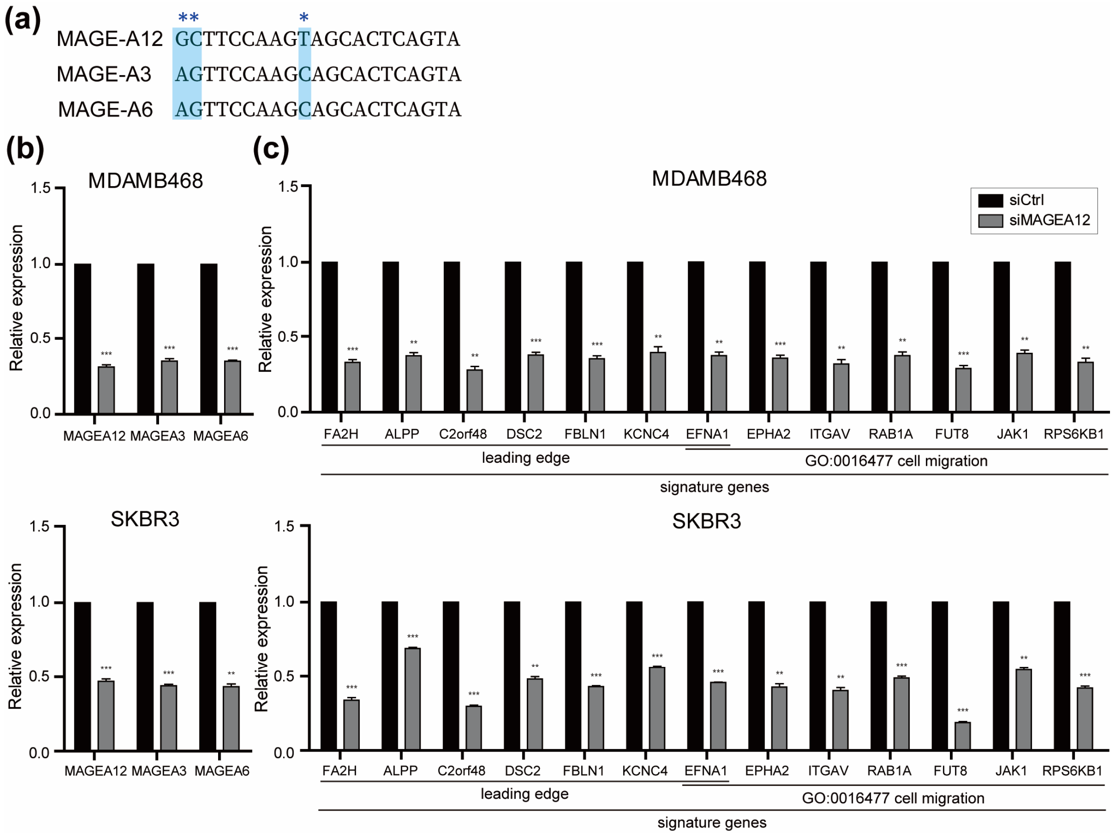

Figure S1. Validation of MAGEA12 siRNA treatment. (a) Target sequence of siRNA against MAGEA12 and the sequence differences between MAGEA12 and MAGEA3, MAGEA6. (b) The expression level of MAGEA12, MAGEA3, and MAGEA6 in MAGEA12 knockdown cells using siRNA. (c) Expression level of selected target genes in MDAMB468 and SKBR3 treated with MAGEA12 siRNA. (** p-value < 0.001, *** p-value < 0.0001)

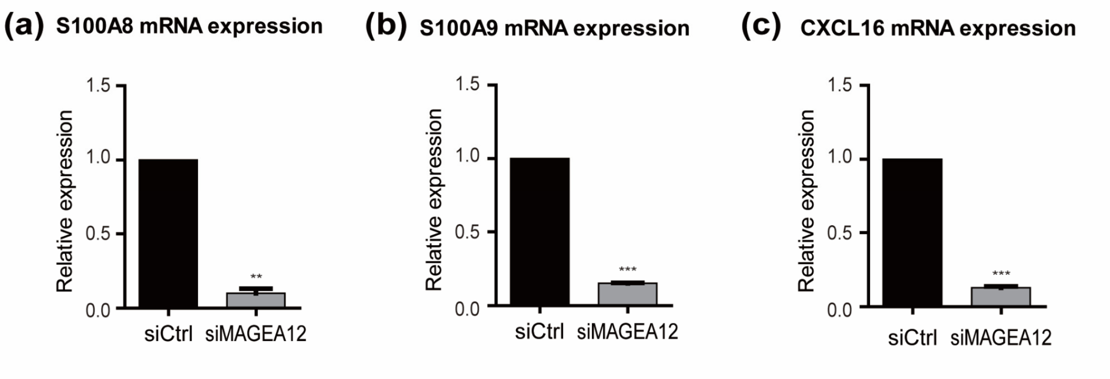

Figure S2. Confirmation of MAGEA12 target gene expression. The expression level of (a) S100A8, (b) S100A9, and (c) CXCL16 in MAGEA12 knockdown cells by qRT-PCR. (** p-value < 0.001, *** p-value < 0.0001)

© 2021 by the authors. Licensee MDPI, Basel, Switzerland. This article is an open access article distributed under the terms and conditions of the Creative Commons Attribution (CC BY) license (http://creativecommons.org/licenses/by/4.0/).
